# The Self-Healing Capacity of the Human Dental Pulp

**Published:** 1903-09

**Authors:** W. D. Miller

**Affiliations:** Berlin, Germany


### International Dental Journal.

Vol. XXIV. September, 1903. No. 9.

### Original Communications.[1]

### THE SELF-HEALING CAPACITY OF THE HUMAN DENTAL PULP.

BY W. D. MILLER, M.D., D.D.S., BERLIN, GERMANY.

It is a fact pretty generally known that in the case of certain animals the dental pulp may undergo reparative processes similar to those which take place in other soft tissues. These reparative processes are frequently observed in teeth having large pulps which are open at the base instead of being everywhere surrounded by narrow unyielding walls, as is the case in human teeth.

It is particularly the pulp of the elephant's tusk which furnishes us the most frequent examples of self-repair, and at any ivory-turner's we may find numbers of specimens illustrating this fact. The question is discussed at length in a series of articles entitled "Studies on the Anatomy and Pathology of the Tusks of the Elephant," beginning in the May number of the *Dental Cosmos* for 1890.

It would not be admissible, however, to conclude that the pulp

[1] The editor and publishers are not responsible for the views of authors of papers published in this department, nor for any claim to novelty, or otherwise, that may be made by them. No papers will be received for this department that have appeared in any other journal published in the country.

of the human tooth must possess the same reparative powers as does that of the elephant. It is, in the first place, devoid of lymphatics which everywhere else perform a very important part in taking up and removing pathological products; but especially it is so small that, being surrounded by unyielding walls, the minutest inflammatory focus must result in intense pressure and stagnation, with a marked tendency to breaking down of the tissue or extending of the inflammation, unless the pressure is relieved by a free exposure. It is for this reason that an abscess or centre of inflammation the size of a pin-head in the pulp of a human tooth may cause excruciating pain, while its presence on the surface of the body might escape notice altogether.

We may accordingly examine hundreds of extracted teeth without coming across a single case of self-repair such as is so commonly met with in ivory. Indeed, it has been very much doubted whether a suppurating pulp could or did ever of itself return to a healthy condition again, and, so far as I am aware, the first case of this kind was described by Gysi in the *Schweizerische Vierteljahrsschrift für Zahnheilkunde,* 1900, page 254. In this case an abscess in the horn of the pulp of an upper first molar had undergone an isolating process, the pulp shutting it off by throwing up a wall of irregular dentine followed by normal dentine.

A second case, in which an abscess cavity having the greatest similarity to those found in ivory had been isolated or encysted, was reported by me in the *Dental Cosmos,* 1901, page 853. In these cases the pulp has protected itself by throwing up a wall of calcific matter (followed by regular dentine), thus shutting off or encysting the offending part.

Since then two other cases have come under my notice, which are illustrated in Figs. 1 and 2. Both these teeth were found among a collection of teeth of unknown history. In the first case (Fig. 1) an upper bicuspid has been attacked by caries on the *approximal surface* and the buccal horn encroached upon. The pulp succeeded in throwing up a sufficient amount of calcific matter at *a* to isolate the diseased horn. In the second case (Fig. 2) we have a lower molar in which the enamel has been completely destroyed by decay and the dentine decalcified nearly or quite to the pulp. The caries, for some reason, stopped at this stage and the decalcified dentine became hard and black. In other words, it is a case of self-healing of the dentine. Strangely enough, we find a self-reparative process going on in the pulp at the same time. It had succeeded in covering its whole surface with a layer of secondary dentine, except at the apex of the one horn, which shows an abscess-cavity the size of a pin-head. This pulp was engaged in encysting at the time of the extraction, having already thrown up a wall of calcific matter at *a,* thus shutting out the abscess, and it would soon have been in a perfectly healthy and normal condition if the tooth had not been unnecessarily extracted.

These cases are beyond a doubt exceedingly rare, and yet they show us that even an abscessed pulp may, without any interference from without, return to a healthy condition, and they certainly justify the inference that a skilful dental practitioner ought to be able to save a certain percentage even of abscessed pulps. And yet all our experience in the conservative treatment of the dental pulp goes to show that there is very little hope of being able to bring about a permanently healthy state in a pulp in which suppuration has once taken place, and that perhaps with very rare exceptions it is better to devitalize such pulps at once. It is, on the other hand, however, going too far when we advocate the devitalization of pulps which manifest only symptoms of beginning local inflammation, or even of perfectly healthy pulps which have been exposed by accident in excavating.

Hyperæmia and fresh cases of slight local inflammation of the pulp may be reduced with tolerable certainty by a judicious use of oil of cloves, thymol, hydronaphthol, nitrate of silver, etc. The latter has proved a very valuable remedy to me in these cases, applied in form of powder on a pledget of cotton moistened in oil of cloves or carbolic acid and sealed in with oxysulphate of zinc. It may be left in the cavity for twenty-four hours, or even longer where the caries has not approached too near the pulp. In two different cases, where the pulps were protected by only a very thin layer of decalcified dentine, I found some months later that they had suffered a painless death without giving any disturbance whatever. I could not attribute the death with certainty to the action of the nitrate of silver, though it is well to avoid an excess in such cases.

Where a healthy pulp is exposed in excavating, the attempt should always be made to save it. After the cavity has been thoroughly cleaned it should be sterilized by the application of non-irritating antiseptics and the pulp capped immediately, care being taken that it does not become unnecessarily infected by access of saliva or use of unclean instruments, and that its surface does not become dried by long exposure to the air. As capping material everything should be rejected which does not adapt itself perfectly to the surface of the pulp without the use of the least pressure. I invariably use oxysulphate of zinc, mixing it to a thin paste. With an instrument with which I can easily reach the surface of the pulp I take up a quantity the size of a large pin-head and bring the paste, not the instrument, into contact with the surface of the pulp, when, if it is of the proper consistency, it will flow off the point of the instrument and spread out over the exposure.

If it is deemed desirable a small quantity of finely pulverized thymol may be mixed with the cement in order to secure a slight permanent antiseptic action. I have made use of this method for fifteen years at the Dental Institute of the University of Berlin, and have been surprised to see how seldom it fails, even in the hands of students. Of course, one must work quickly with the oxysulphate; the moment it begins to harden it ceases to flow, and is then absolutely unfit for capping purposes. I find this method better than that of applying the paste by means of a cap, as the latter cannot be adjusted without a certain amount of pressure, and one cannot see what is taking place under the cap. After the pulp has been protected by the first thin layer of the oxysulphate there can be no objection to then applying the cap, but a layer of oxyphosphate serves the purpose better when it is desired to fill immediately with any material requiring much pressure for its insertion. As a rule, I allow a year to intervene between the capping of an exposed pulp and the insertion of the permanent filling.

